# Comparative Proteomic Analysis of Molecular Differences between Leaves of Wild-Type Upland Cotton and Its *Fuzzless*-*Lintless* Mutant

**DOI:** 10.3390/molecules24203769

**Published:** 2019-10-19

**Authors:** Liping Zhu, Bowen Zheng, Wangyang Song, Chengcheng Tao, Xiang Jin, Hongbin Li

**Affiliations:** 1 College of Life Sciences, Key Laboratory of Xinjiang Phytomedicine Resource and Utilization of Ministry of Education, Shihezi University, Shihezi 832003, China; zhuliping0903@163.com (L.Z.); zhengbw0609@163.com (B.Z.); swywinner@163.com (W.S.); taocc_124@163.com (C.T.); 2 Ministry of Education Key Laboratory for Ecology of Tropical Islands, College of Life Sciences, Hainan Normal University, Haikou 571158, China

**Keywords:** proteomics, cotton leaves, *fuzzless*-*lintless* mutant, gossypol, chlorophyll

## Abstract

*Fuzzless-lintless* mutant (*fl*) ovules of upland cotton have been used to investigate cotton fiber development for decades. However, the molecular differences of green tissues between *fl* and wild-type (WT) cotton were barely reported. Here, we found that gossypol content, the most important secondary metabolite of cotton leaves, was higher in *Gossypium hirsutum* L. cv Xuzhou-142 (Xu142) WT than in *fl*. Then, we performed comparative proteomic analysis of the leaves from Xu142 WT and its *fl*. A total of 4506 proteins were identified, of which 103 and 164 appeared to be WT- and *fl*-specific, respectively. In the 4239 common-expressed proteins, 80 and 74 were preferentially accumulated in WT and *fl*, respectively. Pathway enrichment analysis and protein–protein interaction network analysis of both variety-specific and differential abundant proteins showed that secondary metabolism and chloroplast-related pathways were significantly enriched. Quantitative real-time PCR confirmed that the expression levels of 12 out of 16 selected genes from representative pathways were consistent with their protein accumulation patterns. Further analyses showed that the content of chlorophyll a in WT, but not chlorophyll b, was significantly increased compared to *fl*. This work provides the leaf proteome profiles of Xu142 and its *fl* mutant, indicating the necessity of further investigation of molecular differences between WT and *fl* leaves.

## 1. Introduction

Upland cotton provides the major source of natural fiber materials for the textile industry. Cotton fiber is a single-cell trichome derived from the seed epidermis, and thus is commonly used as an ideal model to investigate plant cell elongation [1]. The development of the cotton fiber cell is commonly defined as four distinct and overlapping stages: fiber initiation, fast elongation, secondary wall biosynthesis, and maturation [2]. Cotton fibers are also characterized as two types: fuzz and lint. Fuzz fibers are initiated after 5 days post anthesis and are shorter than 5 mm at the maturation stage [3]. Lint fibers form the major part used for textile products, initiating before the day of anthesis and finally forming the mature long fibers [4]. To investigate essential molecular events for regulating cotton fiber development, *fuzzless* and/or *lintless* mutants for fiber have been used as negative references for a long time. Many functional genes were identified in upland cotton (*Gossypium hirsutum*) with the help of *fuzzless* and/or *lintless* mutants. Ruan et al. showed that sucrose synthase was essential for cotton fiber initiation using a *fiberless* mutant SL-171 [5]. A single Gly65Val amino acid substitution in actin (GhACT) was identified as the causative mutation of Ligon *lintless Li_1_* short fiber morphology [6]. Novel *Aux/IAA* family genes were cloned and reported significantly upregulated in *linted*-*fuzzless* mutants Xinxiangxiaoji [7]. Along with the development of omics techniques, high-throughput data of *fuzzless* and/or *lintless* cotton provided new insights into the transcriptomic, proteomic, and metabolic differences between the mutants and their wild-type (WT). Transcriptome profiling in Texas Marker 1 (TM-1) cotton and five *fuzzless* mutants was performed to investigate cotton fiber initiation. Genes significantly downregulated in the mutants were mainly protein synthesis-related genes and transcription factors, while genes upregulated in the mutants were DNA/chromatin structure-related genes. Pathway analysis showed that ATP synthesis and sugar and lipid metabolism-related pathways play important roles in fuzz initial development [8]. Comparative transcriptome analysis of WT and its near isogenic *fl* mutant revealed key genes and pathways involved various stages of fiber development. Padmalatha et al. implied the significant role of mitochondria-mediated energy metabolism during the fiber elongation process [9]. Moreover, small RNA deep sequencing of *fuzzless* and/or *lintless* mutants identified differential expression profiles of miRNAs from WT and mutant ovules, which can be expected to regulate transcripts distinctly involved in cotton fiber development [10]. Wang et al. identified the functional lncRNA candidates by differential expression analysis and co-expression network construction during cotton fiber development. Analysis of integrated expression from the sub-genomes of lncRNAs showed that miR397 and its targets were preferably generated by genome polyploidization, indicating their pivotal functions in regulating lignin metabolism in domesticated tetraploid cotton fibers [11]. Sun et al. studied the expression profiles of 54 miRNAs in cotton ovules, fibers, cotyledons, leaves, and flower buds in *G. hirsutum* L. cv. Xuzhou-142 (Xu142) and its *fuzzless*-*lintless* (*fl*) mutant. Thirty-three miRNAs showed different expression during the fiber initiation stage between Xu142 and *fl.* These miRNAs potentially target 723 protein-coding genes, which provides evidence for better understanding of miRNA regulatory roles in the process of fiber development [12].

As mentioned, the *fl* seed mutant that was reported to be a recessive mutation from Xu142 was widely used in genetic and molecular analyses of cotton fiber development for years. The *fl* plant was reported to show no phenotype difference from the WT except the naked seeds’ lack of both fuzz and lint fibers [13,14]. Over the past two decades, numerous regulatory molecules for fiber development had been identified based on comparative transcriptomic, proteomic, and metabolic analyses between Xu142 WT and *fl*, including ethylene signaling, pectic precursor, ascorbate peroxidase, heat shock proteins, linolenic acid, and ROS [15,16,17,18,19,20,21]. Recently, two lncRNAs, XLOC_545639 and XLOC_039050, were characterized for negatively regulating fiber initiation in *fl*, by developing cotton lines with different lint percentages through crossing Xu142 and *fl* [22].

Two-dimensional electrophoresis (2-DE)-based comparative proteomic analyses between WT and *fl* mutant revealed essential regulators for cotton fiber development [23,24]. In addition to cotton fiber, cotton leaves were investigated in many studies to clarify the development process of cotton and its influence on cotton fiber development. Comparative proteomic analyses between transgenic cotton line with a toxin *CrylAc* gene from *Bacillus thuringiensis* (*Bt*) and non-transgenic cotton leaves demonstrated that exogenous DNA in a host cotton genome can affect the plant growth and photosynthesis [25]. Benefiting from the high-throughput mass spectra technique, protein numbers identified within one experiment increased from hundreds (2-DE) to thousands (labeled or label-free LC-MS/MS). In recent years, numerous gel-free proteomic works reported protein expression and post-translational modification changes in cotton leaves under different abiotic stresses, including salt, cadmium, nitric oxide, and cold stresses [26,27,28,29]. For *fuzzless* and/or *lintless* mutants, only one publication described the molecular phenotypes of the leaves of the Ligon *lintless Li_1_* mutant [30]. However, no investigations on the differences between leaves of Xu142 and *fl* have been reported yet.

Gossypol is specifically present in cotton species and plays a crucial role in pathogen and insect defense. As one of the most important sesquiterpene phytoalexins, the gossypol biosynthetic pathway has been characterized recently [31]. Efforts have been made to investigate the molecular mechanism of gossypol, including proteomic and transcriptomic analyses [32]. Chlorophylls are magnesium-tetrapyrrole molecules that play essential roles in photosynthesis. A total of five chlorophylls have been identified: chlorophyll a, b, c, d, and f, of which chlorophyll a and chlorophyll b are the most and second abundant chlorophylls, respectively. The ratio of chlorophyll a to chlorophyll b is important for the regulation of antenna size in photosynthetic organisms, providing higher plants with the ability to optimize their adaptation to varying light conditions [33]. In cotton, chlorophyll a to chlorophyll b ratios were measured in numerous studies on fiber quality and drought tolerance [34,35], however, few works reported the differences of chlorophyll contents between *fiberless* mutant and WT.

For years, Xu142 and fl mutants were widely used for studying the fiber development, but no investigations about the molecular morphologies of leaves were reported. Cotton fiber development is considered a complicated regulation system that is controlled by lots of genes and pathways. We assumed that there must be differences between Xu142 WT and *fl* in tissues other than fibers. In the present work, we found the gossypol content was higher in Xu142 compared with *fl* mutant leaves. Then, we performed a comparative proteomic investigation on the molecular differences between the leaves of Xu142 WT and *fl*, using an improved protein extraction method developed by our group [36]. After digestion by trypsin, LC-MS/MS-based label-free quantitative proteomics analysis was performed. In addition, reverse transcription polymerase chain reaction (RT-PCR) and quantitative real-time PCR (qRT-PCR) assays were used to validate the proteomic data. Bioinformatic analyses indicated that secondary metabolism and chloroplast-related pathways contributed predominantly to the molecular differences between Xu142 WT and *fl*. Therefore, the contents of chlorophyll a and chlorophyll b were further determined. The results for the first time provided insights into the molecular morphologies of leaves of Xu142 WT and *fl*, indicating more complicated molecular regulatory mechanisms of the cotton *fuzzless*-*lintless* mutant.

## 2. Results

### 2.1. Phenotypes and Gossypol Contents of Leaves from Xu142 WT and fl Mutant

**Figure 1:**
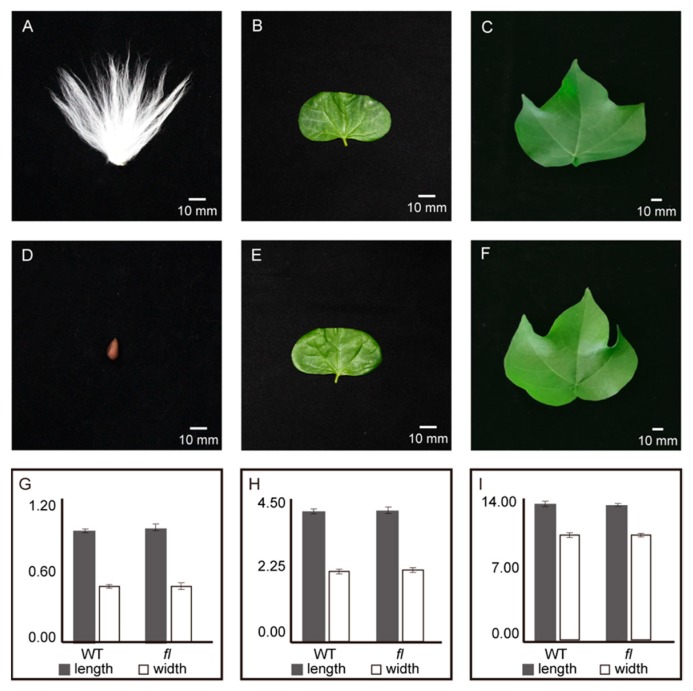
Phenotypes of Xu142 WT and *fl*. The mature seeds (**A**,**D**), cotyledons (**B**,**E**), and mature leaves (**C**,**F**) of Xu142 WT and *fl* mutant are shown. The observation of morphologies of seeds, cotyledons, and mature leaves are shown (**G**,**H**,**I**). The unit of y-axis is centimeter (cm).

The mature seeds of Xu142 WT and *fl* mutant are shown in Figure 1A,D, respectively. No fuzz or lint could be observed on the naked seed of fl. However, the phenotypes of cotyledons and mature leaves had no significant differences between WT (Figure 1B,C) and *fl* (Figure 1E,F). The lengths and widths of mature seeds, cotyledons, and mature leaves were measured and showed no significant differences (Figure 1G–I). This could provide a possible explanation for why scientists were not interested in studying the genetic and molecular differences of leaves between the *fuzzless*-*lintless* mutant and WT.

**Figure 2:**
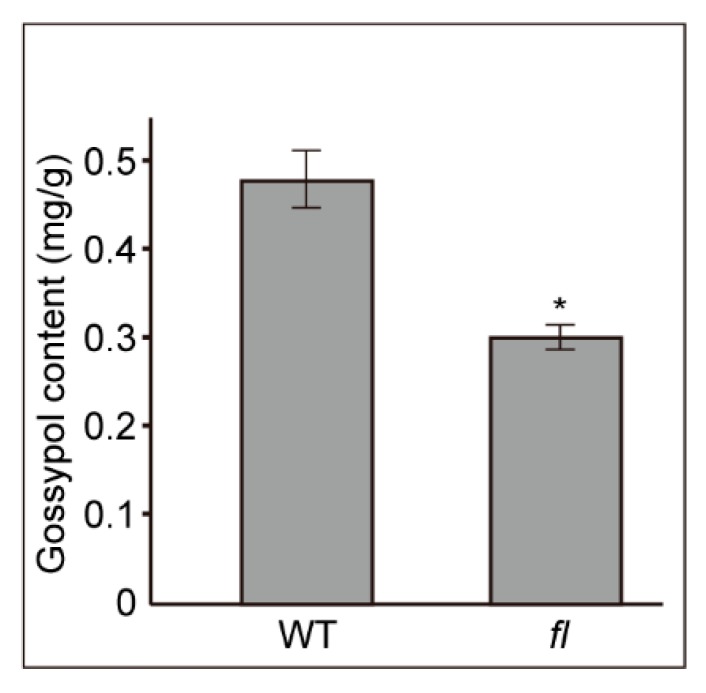
The contents of gossypol from WT and *fl* leaves. n = 5. *, *P* < 0.05.

Gossypol is the most important secondary metabolite of cotton leaves, so we measured the difference of gossypol contents between Xu142 WT and fl mutant leaves. The results indicated that there were significant (P < 0.05) differences in gossypol contents between the two kinds of cotton leaves. The gossypol content was 0.48 ± 0.01 mg/g fresh weight in WT leaves, significantly higher than that of fl (0.30 ± 0.03 mg/g) (Figure 2).

### 2.2. Identification and Validation of WT- and fl-Specific Proteins

**Figure 3:**
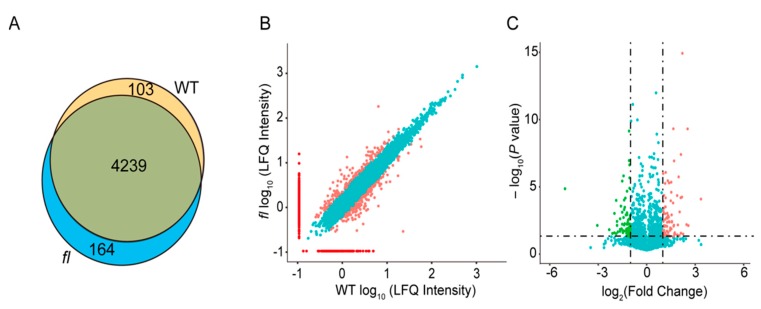
Identification of WT- and *fl*-specific and preferentially accumulated proteins. (**A**) Venn diagram of all proteins identified in WT and *fl*. (**B**) Scattered plots of all identified proteins. The X- and Y-axis represent log_10_ of label-free quantitative intensities of WT and *fl*, respectively. Red, WT- and *fl*-specific proteins; pink, WT and *fl* preferentially accumulated proteins; cyan, proteins showed similar expression levels in both WT and *fl*. (**C**) Volcano plots of all common-expressed proteins. The X- and Y-axis represent log_2_ of fold change and log_10_ of *P* value, respectively. Pink, proteins significantly preferentially accumulated in WT; green, proteins significantly preferentially accumulated in *fl*; cyan, proteins showed no significant differences.

The raw data of mass spectrometry were submitted to the *G. hirsutum* genome database downloaded from Phytozome (v12.1) using Maxquant 6.0 [37]. The raw data along with the searching results have been deposited to the ProteomeXchange (http://proteomecentral.proteomexchange.org) under the dataset identifier PXD014935. A total of 4506 proteins were identified under the criteria of 95% confident, peptides no less than two, and presented in at least two biological replicates. The Venn diagrams for three independent replicates for WT and *fl* leaves are shown (Supplementary Figure S1). Of the 4506 identified proteins, 4239 were common expressed in both WT and *fl* leaves, while 103 were specifically expressed in WT and 164 in *fl* (Figure 3A, Supplementary Tables S1 and S2). Scattered plots of all identified proteins using their log_10_ of label-free intensities showed that most of the common-expressed proteins had no significant changes (cyan spots in Figure 3B). The red spots represent WT- and *fl*-specific proteins and pink spots represent WT and *fl* preferentially accumulated proteins. Volcano plots of 4239 common-expressed proteins showed significantly accumulated proteins (fold change ≥2 and *P* < 0.05) in WT (pink spots in Figure 3C) and *fl* (green spots in Figure 3C). Detailed information for the WT and *fl* accumulated proteins are provided (Supplementary Tables S3 and S4).

**Figure 4:**
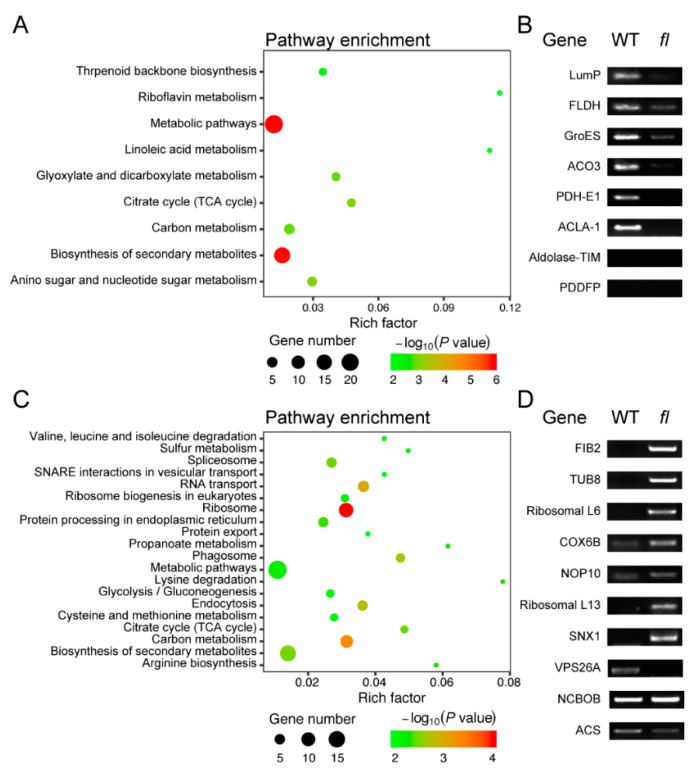
The pathway enrichment analysis of 103 WT- (**A**) and 164 *fl*-specific (**C**) proteins and reverse translation PCR analysis of representative transcripts of WT- (**B**) and *fl*-specific (**D**) proteins. The detailed information of genes and the primers are listed in Supplementary Table S5A.

The Gene Ontology (GO) annotation of 103 WT- and 164 *fl*-specific proteins showed that 103 WT-specific proteins were mainly distributed into chloroplast envelopes and plant-type cell walls for the cellular component category, which is significantly different from that of *fl* (Supplementary Figure S2A). Similar results were obtained for molecular function and biological process categories (Supplementary Figure S2B,C). Kyoto Encyclopedia of Gene and Genomes (KEGG) is a bioinformatics resource for linking genomes to life and the environment. Pathway analysis based on KEGG enrichment analysis showed that the top three enriched pathways for 103 WT-specific proteins were riboflavin metabolism, linoleic acid metabolism, and citrate cycle, while for 164 *fl*-specific proteins, they were lysine degradation, propanoate metabolism, and arginine biosynthesis (Figure 4A,C). It was indicated that more secondary metabolism pathways were enriched for WT-specific proteins while more amino acid metabolism pathways were enriched for *fl*-specific proteins. Together, GO and KEGG analysis of WT- and *fl*-specific proteins showed that there were significant proteome differences between WT and *fl* leaves, although no significant phenotype differences could be observed.

Further, 8 WT-specific and 10 *fl*-specific proteins were randomly selected to perform reverse-transcription PCR validation of proteomic data. The PCR products of six WT-specific and seven *fl*-specific proteins were consistent with the proteomics data, however, two WT-specific proteins showed no PCR products (Aldolase-TIM; PDDFP) and one *fl*-specific protein (NCBOB) showed identical expression levels. There were two *fl*-specific proteins (VPS26A; ACS) showed opposite accumulation levels in gene expression (WT-specific), indicating the complicated post-transcriptional regulation for these two genes (Figure 4B,D). Detailed information for the selected genes and PCR primers are provided (Supplementary Table S5A).

### 2.3. Analysis and Validation of Differential Abundant Proteins (DAPs)

**Figure 5:**
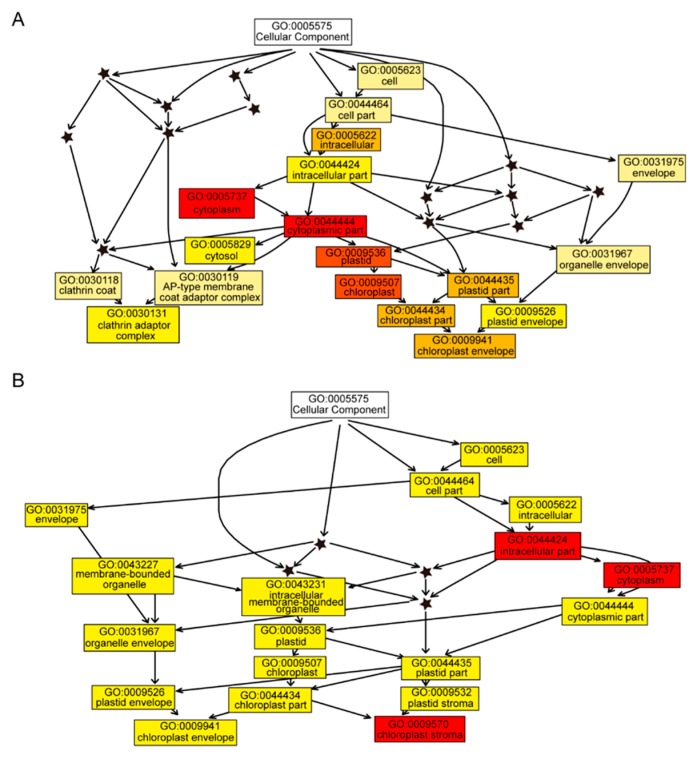
Gene ontology enrichment analysis of 80 WT (**A**) and 74 *fl* (**B**) preferentially accumulated proteins for cellular component category. The GO IDs and descriptions are shown in the boxes. White boxes represent GO category terms. Colored boxes represent significant GO terms, saturation (from yellow to red) of which represents the adjusted *P* value of corresponding GO terms. Nonsignificant GO terms are represented by asterisks.

The WT- and *fl*-specific proteins represented the extreme up- or downregulation of particular proteins. According to the label-free quantification (LFQ) intensities, 80 WT and 74 *fl* preferentially accumulated proteins were also identified (Supplementary Tables S3 and S4). The GO enrichment analysis of the DAPs was performed using online software PlantRegMap (http://plantregmap.cbi.pku.edu.cn/go.php) [38]. The tree view of 80 WT preferentially accumulated proteins for cellular component category showed that clathrin adaptor complex (GO:0030131) and chloroplast envelope (GO:0009941) were significant branch-end terms (Figure 5A). For the 74 *fl* preferentially accumulated proteins, the branch- end terms were chloroplast envelope (GO:0009941) and chloroplast stroma (GO:0009570) (Figure 5B). It looks like the chloroplast-related cellular component GO terms were preferentially enriched by DAPs, indicating molecular differences might be found between WT and *fl* chloroplast. The tree views of DAPs for biological process (Supplementary Figure S3) and molecular function (Supplementary Figure S4) are also provided. The KEGG pathway enrichment analysis was also performed for DAPs (Supplementary Figure S5).

**Figure 6:**
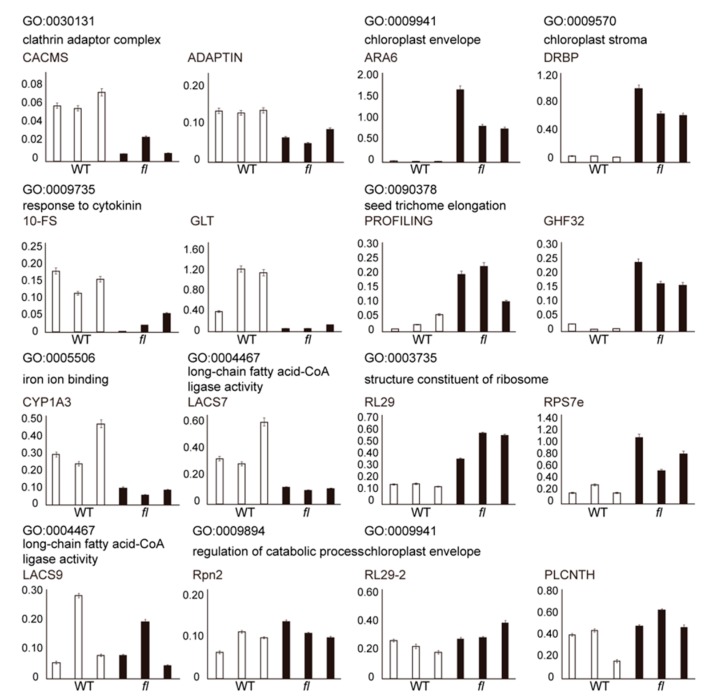
Quantitative real-time PCR validation of representative transcripts selected from nine GO terms of branch-end in the tree view of GO enrichment results. Three independent biological replicates were performed for each transcript and three technical replicates were performed for each biological replicate. The Y-axis represents the relative expression levels of each transcript compared to internal reference gene *GhUBQ*. Detailed information of transcripts and primers is provided in Supplementary Table S5B.

To validate the transcript expression levels of DAPs from branch-end GO terms, qRT-PCR assays were performed for 16 protein-coding genes selected from nine representative branch-end GO terms. A total of 12 transcripts showed consistent expression patterns with proteomic data (Figure 6), while the other four genes showed no significant differences. Three independent biological replicates and three technical repeats were performed. Detailed information for the selected genes and qPCR primers are provided (Supplementary Table S5B).

**Figure 7:**
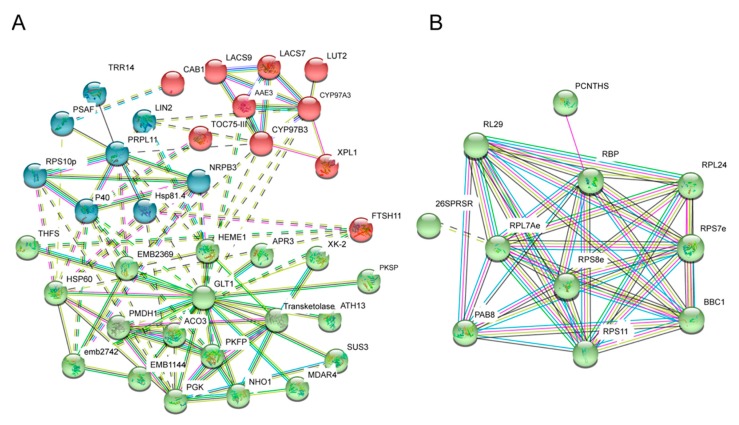
Protein–protein interaction network analysis of 80 WT (**A**) and 74 *fl* (**B**) preferentially accumulated proteins. Protein symbols of network nodes are shown. Different colors represent different clusters of PPI.

Protein–protein interaction (PPI) network analysis could reflect the key elements for a protein dataset. To investigate the network of WT and *fl* preferentially accumulated proteins, the online database STRING (https://string-db.org/) was used. Three network clusters could be observed for the 80 WT preferentially accumulated proteins, represented by different colors (Figure 7A). Only one cluster was recognized for the 74 *fl* preferentially accumulated proteins, mainly composed of ribosome-related proteins (Figure 7B). Moreover, PPI analysis of 103 WT- and 164 *fl*-specific proteins showed that no well-constructed network could be observed for the WT-specific proteins, while three clusters could be recognized for the 164 *fl*-specific proteins, mainly composed of ribosome-related proteins (Supplementary Figure S6).

### 2.4. Determination of Chlorophyll Contents

**Figure 8:**
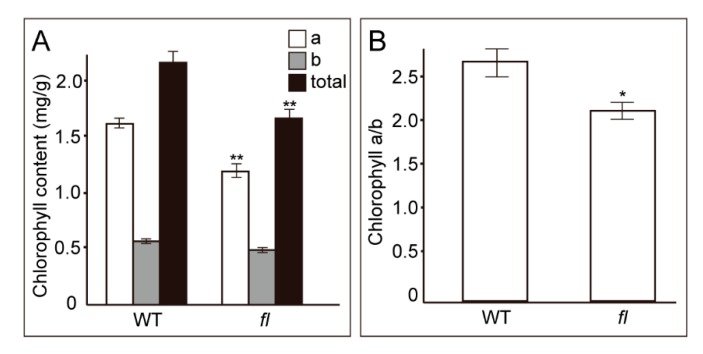
The contents of chlorophyll from WT and *fl* leaves (**A**). The ratios of chlorophyll a to chlorophyll b were calculated (**B**). For all experiments, n = 5. *, *P* < 0.05; **, *P* < 0.01.

The KEGG pathway and GO enrichment analyses, accompanied by PPI results, suggested that secondary metabolism and chloroplast-related pathways might be the most significant molecular differences between WT and *fl*. Thus, the chlorophyll contents were determined in WT and *fl* leaves to validate the reliability of the comparative proteomics data. The chlorophyll content assay showed that the contents of chlorophyll a were significantly lower in *fl*, while the contents of chlorophyll b were equal in both WT and *fl* (Figure 8A). Because the absolute amount of chlorophyll a was higher than chlorophyll b, the total chlorophyll contents and the ratio of chlorophyll a/b were then determined, showing as significantly lower in *fl* than in WT (Figure 8A,B). These data suggested that, despite the coincident extrinsic phenotypes between WT and *fl* leaves, the intrinsic molecular phenotypes were significantly different between WT and *fl* at both proteomic and metabolic levels.

## 3. Discussion

Since the *fl* mutant of Xu142 was found, numerous studies on cotton fiber development were performed, however, no researchers noticed the different molecular phenotypes of leaves between WT and *fl*. Two pairs of recessive genes were reported to control the *fl* phenotype, suggesting there might be some hard-to-find phenotypes in *fl*, such as molecular phenotypes. However, due to the uselessness of the *fl* mutant for the fiber industry, no research focused on such potential invisible phenotypes in nonfiber tissues. Leaves of cotton have various biotic and abiotic stresses which could impact the cotton fiber yield [25,26,27,28,29]. This work is the first one to investigate the molecular phenotype differences between the leaves of Xu142 WT and the *fl* mutant. In addition to 4239 common-expressed proteins, we identified 103 WT- and 164 *fl*-specific proteins, providing the proteome differences between the leaves of Xu142 WT and *fl* (Figure 3A). Further comparative proteomic analyses identified 80 WT and 74 *fl* preferentially accumulated proteins (Figure 3C). RT-PCR and qRT-PCR analyses were used to validate the proteomic data. Most of the transcripts showed similar expression patterns with the corresponding proteins (Figure 4 and Figure 5). There were several exceptions, which could be caused by post-transcriptional regulation or by false positive results of proteomic data, however, it was widely observed in many proteomic and transcriptomic investigations [22].

In addition to the KEGG pathway enrichment, the GO term distribution of 103 WT- and 164 *fl*-specific proteins showed significantly different patterns. For the molecular function category, the top two GO terms were catalytic activity and oxidoreductase activity for WT, but RNA binding and structural constituent of ribosome for *fl* (Supplementary Figure S2B). For the biological process category, the top two GO terms were regulation of protein metabolic process and post-transcriptional regulation of gene expression for WT, but translation and epigenetic regulation of gene expression for *fl* (Supplementary Figure S2C). The top two GO terms for the cellular component category were cytosol and chloroplast envelope for the 103 WT-specific proteins, but cytosol and multivesicular body for the 164 *fl*-specific proteins (Supplementary Figure S2A). Moreover, GO terms related to chloroplast, ribosome, and secondary metabolism were observed in the enrichment analysis of both KEGG pathway and GO (Figure 4 and Figure 5, Supplementary Figures S3–S5). These data, for the first time, determined the molecular differences between WT and *fl* leaves, indicating biological processes related to chloroplast, ribosome, and secondary metabolism could be different in WT and *fl*. The gossypol and chlorophyll content assays validated the bioinformatics analyses (Figure 2 and Figure 8). However, there were some other GO terms and related processes that had been previously reported to be important for cotton development, such as fatty acid metabolism (Supplementary Figures S4 and S5), seed trichome elongation (Supplementary Figure S3), and biosynthesis of amino acids (Supplementary Figures S3 and S5) [39,40,41]. Experiments should be performed in the future to determine whether these pathways differ between the leaves of Xu142 WT and *fl*. Besides, the envelope in the cellular component category, which was enriched in 80 WT preferentially accumulated proteins, was also enriched in other cotton leaves proteomic work [25], indicating the chloroplast-related proteins may vary in different cotton varieties.

Our data for the first time investigated the molecular phenotype differences between the leaves of Xu142 WT and *fl*. We confirmed that, in addition to the loss of fibers, the *fl* mutant showed significantly lower levels of the contents of gossypol and chlorophyll a (Figure 2 and Figure 8). Although the exact gene loci leading to the *fl* mutant phenotype have not been identified yet, we observed some important molecular differences between WT and *fl*, suggesting the gene loci have influences on many metabolism processes involved not only in fiber development but also in cotton leaves. Because of its toxicity, the gossypol was very important for the development of cotton seeds that are widely used as animal feed and the raw material for seed oil as a kind of cooking oil [42,43]. The gossypol content is correlated with number of glands in cotton leaves. However, the gland numbers in Xu142 WT and *fl* showed no differences. Thus, further study on the gossypol-related biological process using Xu142 WT and *fl* leaves will be helpful to understand the gossypol functions. Cotton fiber development is a very complicated process and involves lots of genes and pathways. The *fl* mutant might consume less energy than WT due to loss of fiber tissues, which could be a possible explanation for the lower chlorophyll content in *fl*. Moreover, Xu142 WT and *fl* leaves could be ideal objects for studying the fatty acid, trichome, and ribosome-related biological processes in cotton species.

In summary, to date, no comparative research has been reported between Xu142 WT and *fl* leaves. This work for the first time performed proteomic analysis to investigate the molecular phenotype differences between Xu142 WT and *fl* leaves. A total of 103 WT- and 164 *fl*-specific proteins, and 80 WT and 74 *fl* preferentially accumulated proteins were identified. RT-PCR and qRT-PCR were used to validate the transcripts levels of representative differential abundant proteins. Bioinformatic analyses showed that chloroplast, secondary metabolism, and ribosome-related pathways were the most significantly enriched KEGG and GO pathways. Further gossypol and chlorophyll contents assays showed that the contents of gossypol and chlorophyll a were significantly reduced in *fl* leaves. Our results provide the leaf proteome profiles of Xu142 and its *fuzzless*-*lintless* mutant, identifying the molecular phenotype differences between WT and *fl*, indicating the necessity of further investigation on WT and *fl* leaves.

## 4. Materials and Methods

### 4.1. Plant Materials and Morphological Observation

Upland cotton *Gossypium hirsutum* L. cv Xuzhou-142 and *fuzzless*-*lintless* mutant were grown in an incubation house under 60% humidity, 34 °C, and a 14 h/10 h light/dark cycle. Leaves were harvested from four-week-old seedlings (3–4 true leaves per plant from 3 to 5 independent plants) from Xu-142 WT and *fl*. The leaves were frozen in liquid nitrogen immediately after harvest, then stored at −80 °C until use.

Stereoscopes were used for morphological observation for leaves from Xu142 and the *fuzzless*-*lintless* mutant.

### 4.2. Gossypol Contents Determination

The determination of gossypol contents was carried out as described [32]. Briefly, 1, 3, 5, 7, 9 μg gossypol (Solarbio, Beijing, China) was dissolved using chloroform as standard solution. The absorbance of the colored solution was measured at 364 nm against the chloroform as a reference, and calibration curves were prepared. Ten grams of fresh leaves were dried at 40 °C for one hour to remove moisture, then ground into powder, and then 250 mg of the ground powder was passed through a 2 mm sieve. After that, 10 mL of chloroform was added to dissolve the powder and was fully whirled for 4 h. After filtering the solution, 1 mL of the liquid was taken up, diluted to 25 mL using chloroform, and the absorbance of the colored solution was measured at 364 nm to determine the gossypol content of cotton leaves. One biological replication contained leaves from three to five independent plants, and five biological replications were performed.

### 4.3. Protein Extraction

An improved protein extraction method [36] was used to extract proteins of leaves from Xu142 and *fl*. Briefly, 1 g of cotton leaves was used for TCA protein extraction method. Then, the dissolved proteins were placed into a new tube, and 3 volumes of BPP buffer were added and vortexed for 10 min at room temperature. Thirdly, 2 volumes of Tris saturated phenol (pH 8.0) were added and centrifuged at 4 °C, 15,000 rpm for 20 min. The supernatant was mixed with 5 volumes of ammonium sulphate super-saturated buffer to precipitate the proteins at −20 °C overnight. Next, it was centrifuged at 4 °C, 15,000 rpm for 20 min, then the pellets were washed twice using prechilled methanol and acetone. Finally, the pellets were air dried and recovered using 100 μL of the lysis buffer for at least two hours and then centrifuged at 12000 rpm for 30 min in room temperature, then the supernatant was transferred to a new centrifugal tube and stored at −80 °C until use. One biological replication contained leaves from three independent plants, and three biological replications were performed.

### 4.4. Protein Identification and Bioinformatic Analysis

The LC-MS/MS analysis was performed as previously described [36]. Briefly, 400 μg of total protein were used for trypsin digestion, and the digestion was performed as previously described. The subsequent LC-MS/MS experiment was carried out on a Triple TOF 5600 plus system (AB Sciex, Shanghai, China) coupled with an UltiMate™ 3000 RSLCnano (Dionex, Thermo, Shanghai, China). The resulting MS spectra were acquired across the mass range of 350–1500 *m*/*z* in high resolution mode (>30,000) and the accumulation time was 50 ms per spectrum. The protein identification and LFQ were analyzed against the downloaded cotton protein database from Phytozome (v12.1) using MaxQuant v1.6.3.4 software [43]. We used the enzymatic rule of trypsin/P with a maximum of two missed cleavages. The main search peptide tolerance was set to 50 ppm and the ion trap MS/MS match tolerance was set to 0.5 Da. Peptide-to-spectrum match level was set at 0.01 FDR with an additional minimal Andromeda score of 40 for modified peptides as these settings are most commonly used by researchers. Protein FDR was set at 0.01 and estimated by using the reversed search sequences. We performed label-free quantitation with MaxQuant’s standard settings.

The LFQ results, accompanied by raw MS files, were uploaded to the ProteomeXchange Consortium (http://proteomecentral.proteomexchange.org) under the dataset identifier PXD014935. Potential contaminants and reverse sequences were removed, and proteins with no less than two peptides, *q*-value < 0.05, were considered successfully identified in each set of MS data. The proteins that were identified in at least two biological replicates were finally included in the subsequent analysis. The thresholds for WT and *fl* preferentially accumulated proteins were set as two fold-change with a *P*-value < 0.05. For KEGG enrichment analysis, sequences of specific proteins were submitted to the KOBAS 3.0 (http://kobas.cbi.pku.edu.cn/anno_iden.php) [44]. For GO enrichment analysis, online software PlantRegMap was used (http://plantregmap.cbi.pku.edu.cn/go.php) [37].

### 4.5. RNA Extraction and PCR/qPCR Validation

The total RNA from leaves of Xu142 and *fl* was extracted using an RNAprep Pure Plant Kit (Tiangen, Beijing, China). DNAse treatment was included in the Kit. First-strand complementary DNA (cDNA) was generated using a reverse transcription system (Takara, Kusatsu, Japan) in accordance with the manufacturer’s instructions. An amount of 2 μg RNA was used for reverse transcription. The reverse transcription PCR was performed as previously described [45]. The expression levels of each gene were visualized using agarose gel electrophoresis of the corresponding PCR products. Quantitative real-time PCR was performed using the SYBR green real-time PCR master mixes (Appliedbiosystems, Foster, CA, USA). The 2^−ΔΔ^CT method was used to calculate the relative expression levels of the target genes. We used ubiquitin (*GhUBQ*) as a housekeeping gene for qRT-PCR experiments, and specific primers in this work are provided in Supplementary Table S5. One replication contained leaves from three independent plants, and three biological replications were performed.

### 4.6. Chlorophyll Contents Measurement

Fresh leaves of Xu142 and *fl* from four-week-old seedlings were used to determine chlorophyll contents according to the described method [46]. The 0.2 g leaves were ground thoroughly within 2 mL 80% (*v*/*v*) acetone, then 10 mL acetone was added, before incubating at room temperature in the dark for 30 min. Samples were centrifuged at 15,000 *g* for 15 min to precipitate cellular debris. Supernatant was used to measure the chlorophyll contents by a UV spectrophotometer (Shimadzu UV-160, Kyoto, Japan). Absorbance was measured at 663 nm and 646 nm to measure chlorophyll a and b, respectively. Total chlorophyll a and b was determined using the following formulae: (12.7 × A663 − 2.69 × A646) × Volume/Weight = Chl a mg/g FW and (22.9 × A646 − 4.86 × A663) × Volume/Weight = Chl b mg/g FW. One replication contained leaves from three independent plants, and five biological replications were performed.

## Supplementary Materials

The following are available online at https://www.mdpi.com/1420-3049/24/20/3769/s1, Figure S1: Venn diagram of the proteins identified by high-throughput LC-MS/MS from WT and *fl* leaves, Figure S2: The enriched Gene Ontology (GO) terms in cellular component (A), molecular function (B) and biological process (C) and categories for 103 WT- and 164 *fl*-specific proteins in leaves, Figure S3: Tree map of the enriched Gene Ontology (GO) terms in biological process for 80 WT (A) and 74 *fl* (B) preferential accumulated proteins, Figure S4: Tree map of the enriched Gene Ontology (GO) terms in molecular function for 80 WT (A) and 74 *fl* (B) preferential accumulated proteins, Figure S5: The KEGG pathway enrichment analysis of 80 WT (A) and 74 *fl* (B) preferential accumulated proteins, Figure S6: Protein-protein interaction network analysis of 103 WT- (A) and 164 *fl*- (B) specific proteins, Table S1: Detailed information of the proteins specially accumulated in Xu142, Table S2: Detailed information of the proteins preferentially accumulated in *fuzzless*-*lintless* mutant, TbaleS3: Detailed information of the proteins preferentially accumulated in Xu142, TbaleS4: Detailed information of the proteins preferentially accumulated in *fuzzless*-*lintless* mutant, TbaleS5: Primers used in this work.
