# Supplementary material for: Comparative Proteomic Analysis of Molecular Differences between Leaves of Wild-Type Upland Cotton and Its Fuzzless-Lintless Mutant
Source: Molecules. 2019 Oct 19;24(20):3769. doi: 10.3390/molecules24203769 (PMC6832260; doi:10.3390/molecules24203769)
Supplement: Supplementary file 1 [file molecules-24-03769-s001.zip › SupplementaryFigures_20191018.docx]

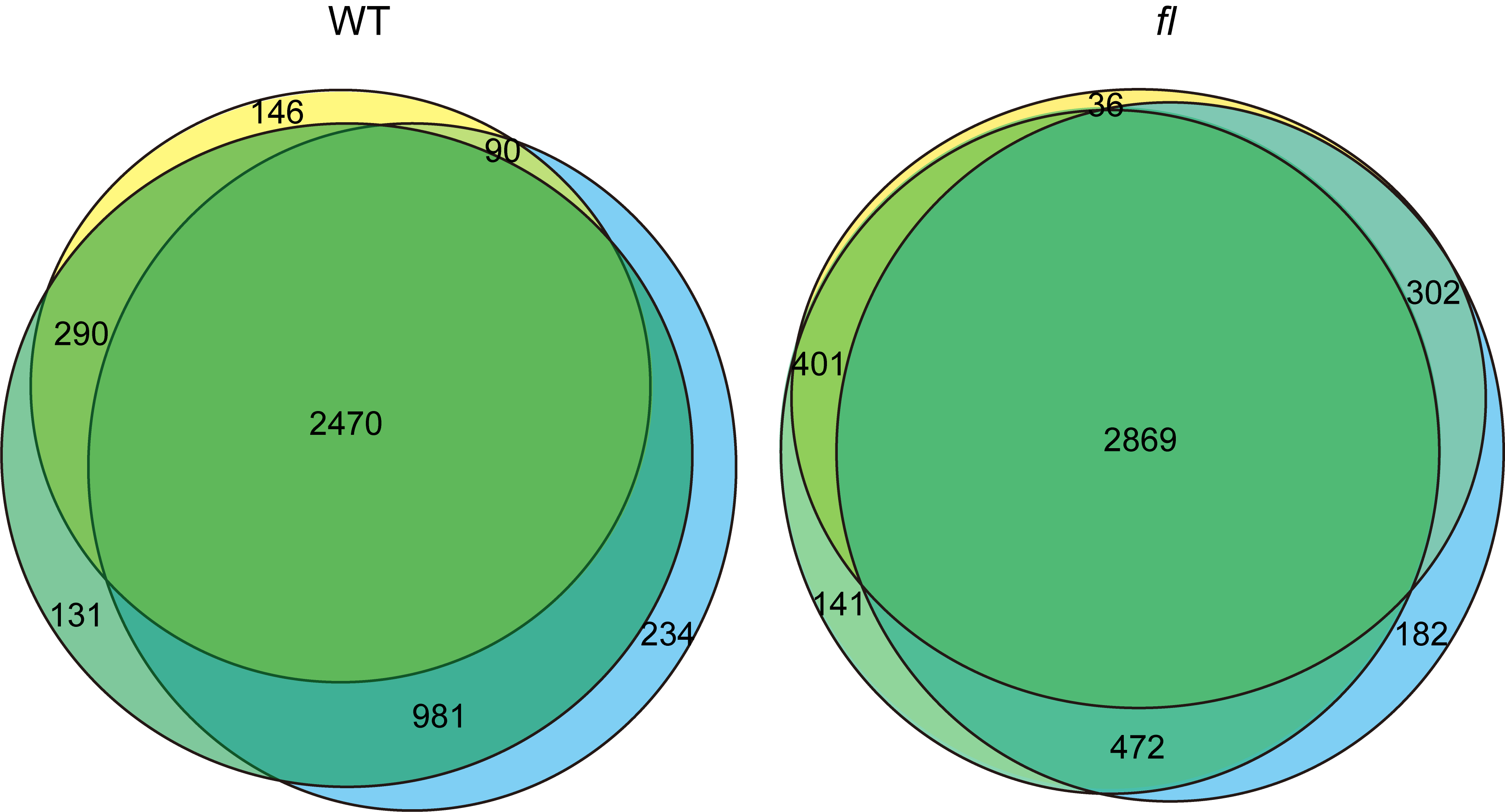


**Figure S1.** Venn diagram of the proteins identified by high-throughput LC-MS/MS from WT and *fl* leaves. The diagram showed the distribution of the protein numbers that identified in three biological replicates.


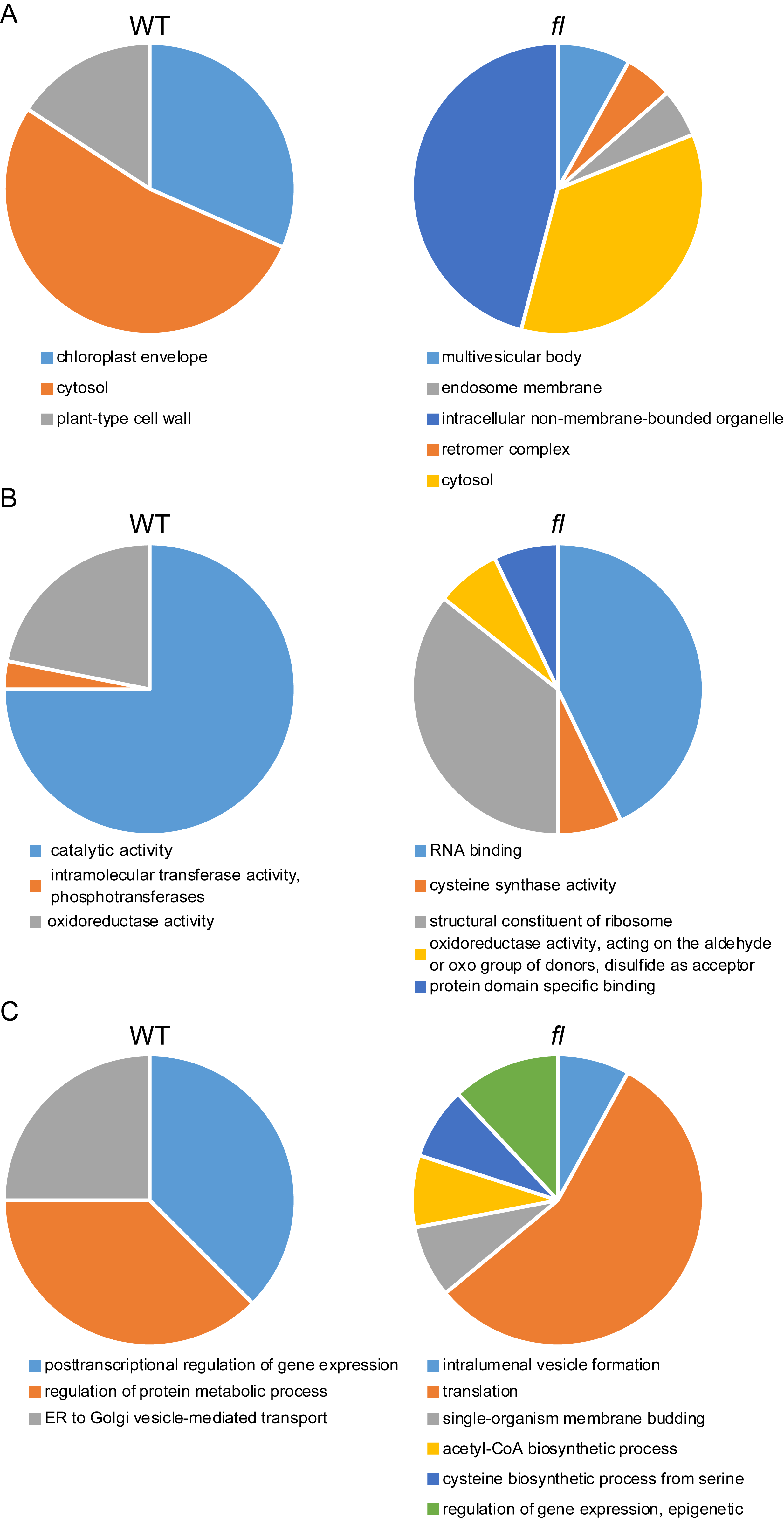


**Figure S2.** The enriched Gene Ontology (GO) terms in cellular component (A), molecular function (B) and biological process (C) and categories for 103 WT- and 164 *fl-*specific proteins in leaves. Different colors represent different categories of GO terms (bottom), and the area size represents the protein content in corresponding category.


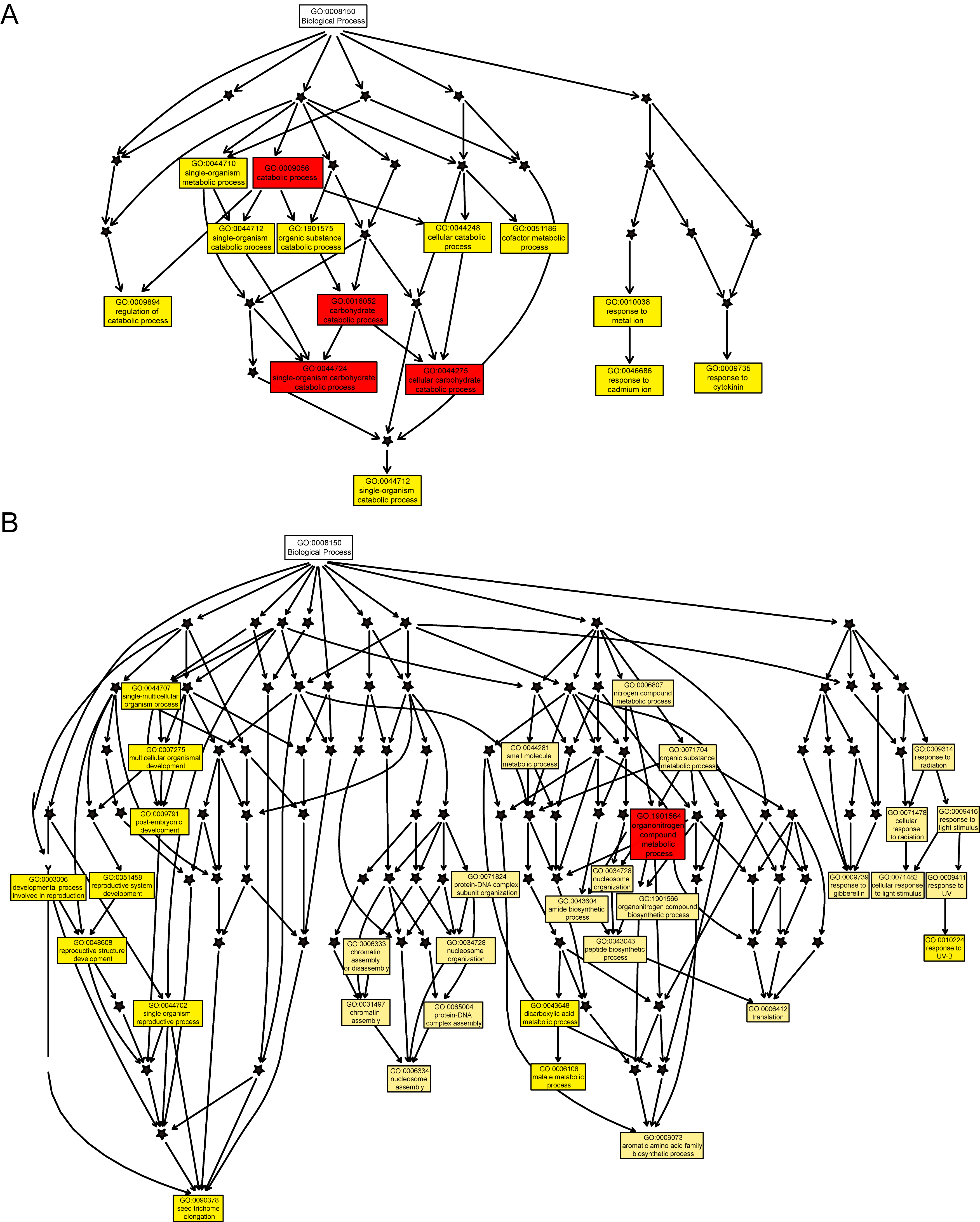


**Figure S3.** Tree map of the enriched Gene Ontology (GO) terms in biological process for 80 WT (A) and 74 *fl* (B) preferential accumulated proteins. The GO IDs and descriptions were shown in the boxes. The saturation of colors (from yellow to red) represents the adjusted *P* value of corresponding GO terms. Non-significant GO terms were represented by asterisks.


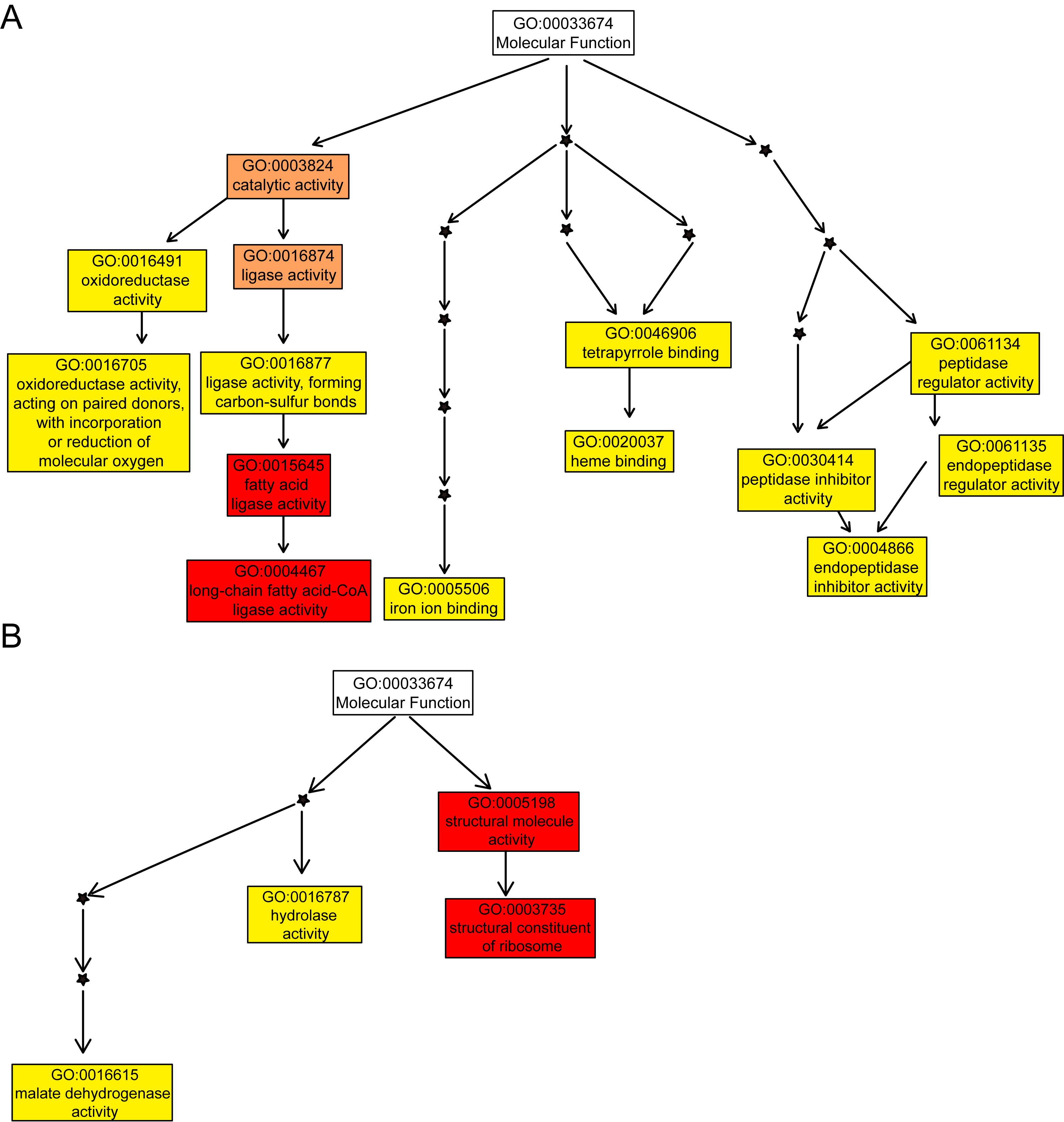


**Figure S4.** Tree map of the enriched Gene Ontology (GO) terms in molecular function for 80 WT (A) and 74 *fl* (B) preferential accumulated proteins. The GO IDs and descriptions were shown in the boxes. The saturation of colors (from yellow to red) represents the adjusted *P* value of corresponding GO terms. Non-significant GO terms were represented by asterisks.


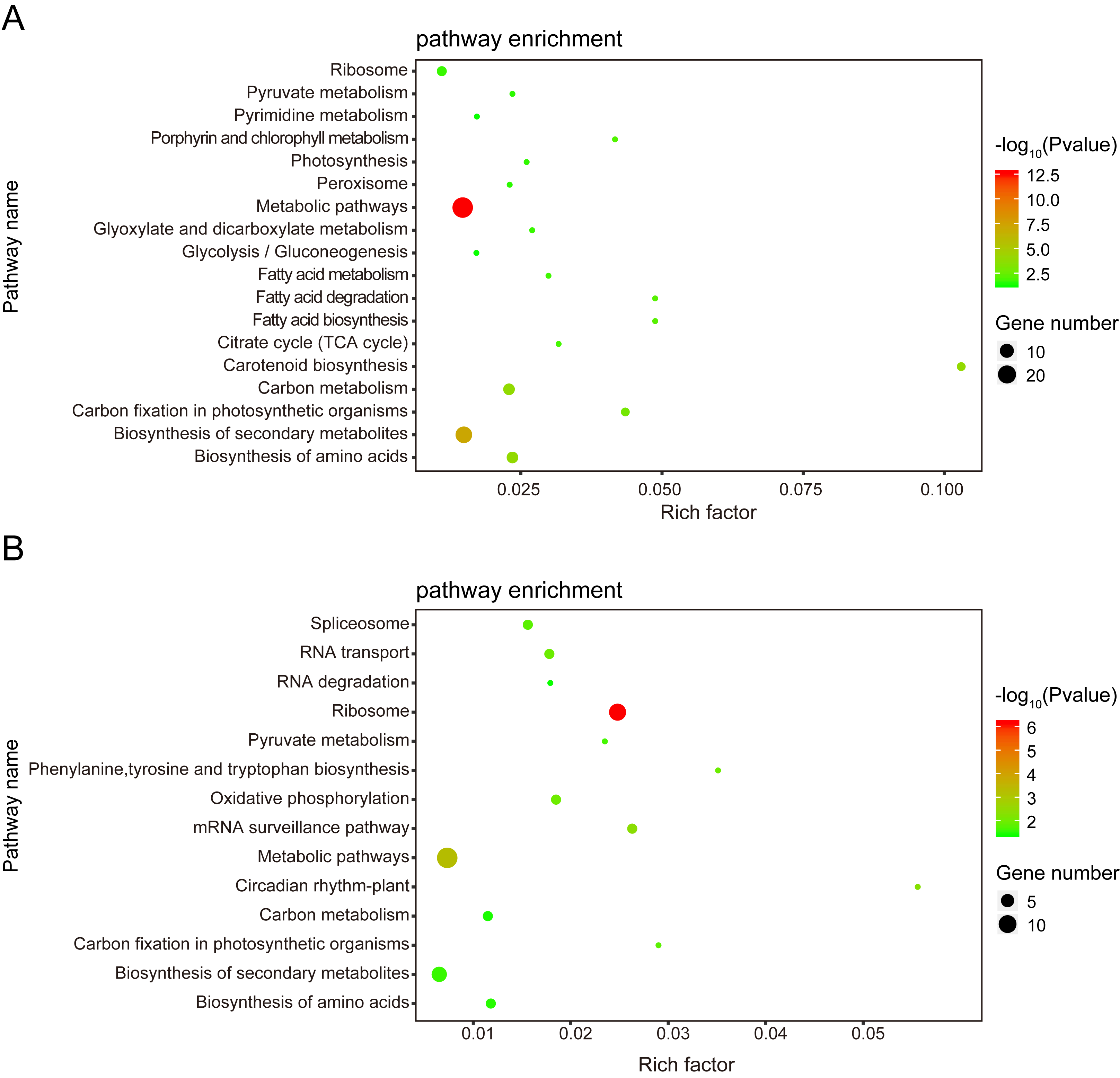


**Figure S5.** The KEGG pathway enrichment analysis of 80 WT (A) and 74 *fl* (B) preferential accumulated proteins.

**
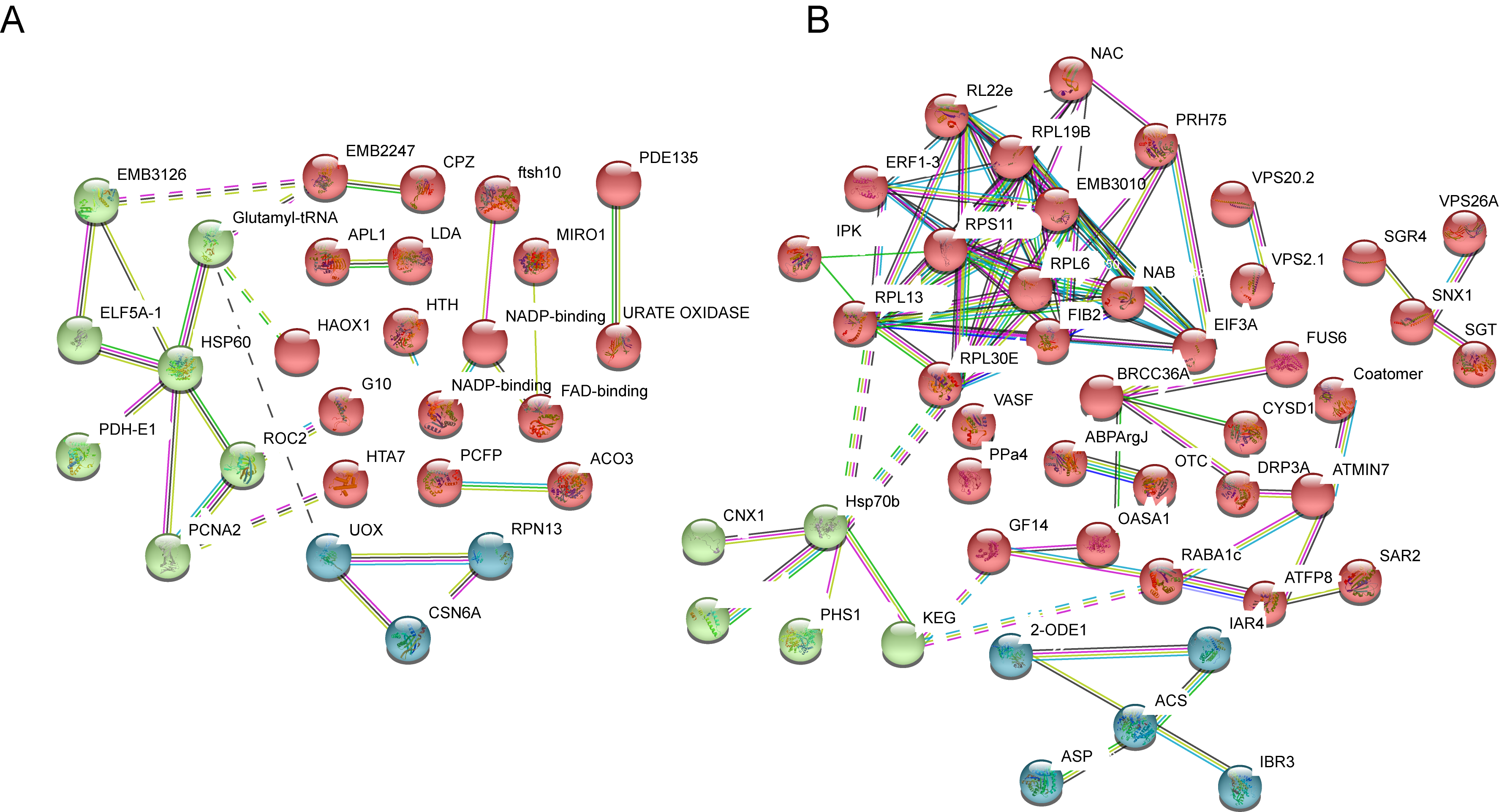
**

**Figure S6.** Protein-protein interaction network analysis of 103 WT- (A) and 164 *fl*- (B) specific proteins. Protein symbols of network nodes were shown. Different colors represent different clusters of PPI.
